# Use of the Wilshire Equations to Correlate and Extrapolate Creep Data of Inconel 617 and Nimonic 105

**DOI:** 10.3390/ma11122534

**Published:** 2018-12-13

**Authors:** Vito Cedro III, Christian Garcia, Mark Render

**Affiliations:** 1 National Energy Technology Laboratory, 626 Cochrans Mill Road, Pittsburgh, PA 15236, USA; 2 Mechanical Engineering Department, University of Texas Rio Grande Valley, 1201 West University Drive, Edinburg, TX 78539, USA; christian.garcia03@utrgv.edu; 3 KeyLogic Systems, Inc., 3168 Collins Ferry Road, Morgantown, WV 26505, USA; mrender@keylogic.com

**Keywords:** Wilshire equation, Larson–Miller parameter, creep strength, Inconel 617, Nimonic 105, time to rupture

## Abstract

Advanced power plant alloys must endure high temperatures and pressures for durations at which creep data are often not available, necessitating the extrapolation of creep life. A recently developed creep life extrapolation method is the Wilshire equations, with which multiple approaches can be used to increase the goodness of fit of available experimental data and improve the confidence level of calculating long-term creep strength at times well beyond the available experimental data. In this article, the Wilshire equation is used to extrapolate the creep life of Inconel 617 and Nimonic 105 to 100,000 h. The use of (a) different methods to determine creep activation energy, (b) region splitting, (c) heat- and processing-specific tensile strength data, and (d) short-duration test data were investigated to determine their effects on correlation and extrapolation. For Inconel 617, using the activation energy of lattice self-diffusion as $Q_{C}^{*}$ resulted in a poor fit with the experimental data. Additionally, the error of calculated rupture times worsened when splitting regions. For Nimonic 105, the error was reduced when heat- and processing-specific tensile strengths were used. Extrapolating Inconel 617 creep strength to 100,000 h life gave conservative results when compared to values calculated by the European Creep Collaborative Committee.

## 1. Introduction

Innovations in power generation require materials that are capable of withstanding high temperatures and stresses for at least 100,000 h of operation time. The high temperatures and pressures found in advanced power plants can induce creep failure in alloys. Consequently, alloys must be tested so that creep failures are avoided during service. However, data regarding creep of new advanced power plant alloys are often not available at times relevant to the required design life. In particular, nickel-based superalloys—promising alloys for ultra-supercritical power plant applications—have no creep rupture data at 100,000 h in the literature. The longest creep rupture test of a nickel-based superalloy known to the authors—an Inconel 617 specimen last reported at 90,936 h—is ongoing and is the result of a joint effort led by the U.S. Department of Energy and the Ohio Coal Development Office [1,2]. The same effort produced creep rupture data for Inconel 617 to 43,706 h (completed), and Inconel 740 to 30,957 h (completed) and 56,550 h (ongoing). In a similar effort, creep rupture data of Nimonic 105 was generated to 34,955 h [3,4]. Hence, extrapolating the creep life of these alloys is necessary to determine if they are suitable for use.

Various methods have been proposed to extrapolate creep life. The Wilshire equations [5] are a recently-developed extrapolation method that has been used to predict long-term creep behavior of high-temperature, creep-resistant alloys [6]. Different approaches have been used to fit the Wilshire equation to creep rupture data. In this article, the Wilshire equation for time to rupture and the Larson–Miller parameter (LMP) equation are used to correlate and extrapolate the creep life of two nickel-based superalloys, Inconel 617 and Nimonic 105. The Wilshire equation’s goodness of fit and the error of the calculated rupture times resulting from the use of different creep activation energy ($Q_{C}^{*}$) values determined by various methods are compared. This article also investigates the effect of splitting creep rupture data into above- and below-yield stress regions, the effect of heat- and processing-specific tensile strength (TS) values, and examines the ability of the Wilshire equation to predict creep life greater than 10,000 h using data with rupture times less than 10,000 h. Additionally, the calculations of the Wilshire and LMP equations are compared. The paper is constructed as follows: Section 2 gives a brief overview of the Wilshire and LMP equations; Section 3 discusses the data sets and methods used to obtain $Q_{C}^{*}$, split stress regions, and how Larson–Miller fitting parameters were obtained; Section 4 outlines the calculations and results of the study; and the final section presents conclusions.

## 2. Wilshire and Larson–Miller Parameter Equations

The classic power law equation, which is a combination of the Arrhenius [7] and Norton [8] equations, is the most established description of the creep of materials. The equation is defined as:

(1) $${\dot{\varepsilon }}_{m}=A\sigma^{n}e^{-Q_{C}/RT},$$

where ${\dot{\varepsilon }}_{m}$ is the minimum creep rate, *A* is a material parameter, *σ* is applied stress, *n* is the stress exponent, *Q_C_* is the creep activation energy, *R* is the universal gas constant, and *T* is absolute temperature. The Monkman–Grant equation [9] is defined as:

(2) $${\dot{\varepsilon }}_{m}{}^{\alpha }\;t_{r}=C_{M},$$

where *t_r_* is the time to rupture, *C_M_* is a constant, and *α* is the slope of $\log t_{r}$ vs. $\log{\dot{\varepsilon }}_{m}$. This equation can be coupled with Equation (1), with *α* set equal to 1, to produce the following time-to-rupture-based version of the classic power law equation

(3) $$t_{r}=\frac{C_{M}}{A\sigma^{n}e^{-Q_{C}/RT}}.$$

However, this equation is unreliable for predicting creep life at temperatures, stresses, and durations at which there are no experimental data available. This is due to the changing and difficult-to-predict stress exponent [10,11], which is a function of stress and temperature. Additionally, creep activation energy is a function of applied stress. Therefore, a creep activation energy that has been calculated in one stress region cannot be extrapolated to another. Many techniques to extrapolate creep life reliably from limited data have been proposed [12,13], including the relatively new Wilshire equation and the well-established Larson–Miller parameter equation.

### 2.1. Wilshire Equation

In 2007, Wilshire and Battenbough [5] developed a physically-based yet fairly simple method to represent creep life as a function of applied stress and temperature in uniaxial tests. The proposed equations are:

(4) $$\frac{\sigma }{\sigma_{TS}}=\exp\left(-k_{1}{\left[t_{r}\exp\left(-\frac{Q_{C}^{*}}{RT}\right)\right]}^{u}\right),$$

(5) $$\frac{\sigma }{\sigma_{TS}}=\exp\left(-k_{2}{\left[{\dot{\varepsilon }}_{m}\exp\left(\frac{Q_{C}^{*}}{RT}\right)\right]}^{v}\right).,$$

(6) $$\frac{\sigma }{\sigma_{TS}}=\exp\left(-k_{3}{\left[t_{\varepsilon }\exp\left(-\frac{Q_{C}^{*}}{RT}\right)\right]}^{w}\right),$$

where $\sigma /\sigma_{TS}$ is the ratio of applied stress to ultimate tensile strength, $t_{r}$ is time to rupture, ${\dot{\varepsilon }}_{m}$ is minimum creep rate, $t_{\varepsilon }$ is time to strain, $Q_{C}^{*}$ is creep activation energy determined at constant $\sigma /\sigma_{TS}$, *R* is the universal gas constant, *T* is absolute temperature, and $k_{1}$, *u*, $k_{2}$, *v*, $k_{3}$, and *w* are fitting constants. Heat- and processing-specific tensile strength values may be used if data were collected from specimens of multiple heats. Applied stress can be normalized by yield strength (*σ_YS_*), but normalization by ultimate tensile strength causes the stress ratio to always lie between zero and one. The boundaries of the Wilshire equations are

$$t_{r}\to 0,\;{\dot{\varepsilon }}_{m}\to \;\infty ,\;t_{\varepsilon }\to 0\;\mathrm{when}\;\frac{\sigma }{\sigma_{TS}}\to 1,\;t_{r}\to \infty ,{\dot{\varepsilon }}_{m}\to \;0,\;t_{\varepsilon }\to \infty \;\mathrm{when}\;\frac{\sigma }{\sigma_{TS}}\to 0.$$

### 2.2. Larson–Miller Parameter Equation

A common method used to extrapolate creep life is the Larson–Miller parameter equation [14], which predates the Wilshire equation by half a century. The LMP equation is:

(7) $$\mathrm{LMP}=T\left(\log_{10}\left(t_{r}\right)+C\right),$$

where *T* is absolute temperature, *C* is a material constant, and $t_{r}$ is time to rupture. The LMP, a function of stress, is often described by the following fitting function.

(8) $$\mathrm{LMP}\left(\sigma \right)=B_{0}+B_{1}\log\left(\sigma \right)+B_{2}\log{\left(\sigma \right)}^{2}+B_{3}\log{\left(\sigma \right)}^{3}+\ldots B_{m}\log{\left(\sigma \right)}^{m}.$$

## 3. Methods

### 3.1. Data

The Wilshire and Larson–Miller parameter equations both require the following data from creep rupture tests: temperature, applied stress, and time to rupture. Additionally, the Wilshire equation requires the material’s ultimate tensile strength at each test temperature. Yield strength values are not included in the Wilshire equations, but according to Wilshire and Battenbough [5] can be used to group high- and low-stress data for analysis. Data were obtained from tables or extracted from plots in various publications using Dagra digitization software (version 2.0.12) [15].

#### 3.1.1. Inconel 617

Inconel 617 tensile and yield strength data were obtained and extracted from a conference presentation [16]. The data is composed of multiple datasets and there is noticeable scatter in the plotted data. A trendline of the average tensile strength of the combined datasets was used for the extraction of data. Creep test data were extracted from five sources [1,17,18,19,20], the longest of which extends to 43,706 h. A total of 420 creep rupture data points were extracted; 386 have a rupture time less than 10,000 h and 354 have an applied stress less than yield strength.

#### 3.1.2. Nimonic 105

For Nimonic 105, tensile and yield strength values and creep rupture data were obtained from two technical reports [3,4] and email correspondence with one of their authors [21]. Specimens with the following processing conditions were tested at temperatures from 760 to 816 °C for up to 34,955 h: as-processed (AP), peak-aged (PA), and over-aged (OA). Heat- and processing-specific tensile and yield strength values are available at 760 and 816 °C. Linear interpolation was used to calculate tensile and yield strength values at intermediate temperatures. Of the 33 specimens, eight were aged at 774 °C for one or two years prior to testing. It is not known which of the eight specimens were aged for each timeframe, so tensile and yield strength values for both timeframes were averaged. Tensile and yield strength values at 760 and 816 °C for each heat and processing condition are shown in Table A1 of Appendix A. All data points have an applied stress less than yield strength and 16 data points have a rupture time less than 10,000 h. The overall scatter of the data is low compared to that of Inconel 617.

### 3.2. Wilshire Equation

Although three versions of the Wilshire equation exist, in this study Equation (4)—the Wilshire equation for time to rupture—is used. Several methods have been used in the literature to determine $Q_{C}^{*}$. In this work, $Q_{C}^{*}$ was determined using multiple methods.

First, $Q_{C}^{*}$ values were determined using Arrhenius plots. This method is the most well-known, and it is assumed [22] that Wilshire used this method in his papers [4,5,23,24,25,26,27,28,29,30,31]. For this approach, existing creep data is regressed and rupture times at constant stress ratios are calculated. This work uses least squares regression and stress ratios at every tenth value (i.e., $\sigma /\sigma_{TS}=$ 0.1, 0.2, 0.3, etc.) with suitable data at each test temperature. Next, an Arrhenius plot of the natural log of time to rupture vs. the inverse absolute temperature is generated. For each stress ratio, a $Q_{C}^{*}$ value is defined as the slope of a line of best fit multiplied by the universal gas constant. An average is then taken of all $Q_{C}^{*}$ values to determine the final $Q_{C}^{*}$ value.

Second, $Q_{C}^{*}$ values were determined by optimizing the correlation of data on a Wilshire plot (ln[*t_r_* exp(−$Q_{C}^{*}$/*RT*)] vs. ln[−ln(*σ/σ_TS_*)]), as performed by Whittaker [32,33,34] and Jeffs [35]. When calculating $Q_{C}^{*}$ using an Arrhenius plot, the delta between the stress ratios influences the final average $Q_{C}^{*}$ value (e.g., $\sigma /\sigma_{TS}\;=$ 0.1, 0.2 and 0.3 compared to $\sigma /\sigma_{TS}=$ 0.1, 0.15, 0.2, 0.25, and 0.3). This presents a concern when calculating an average $Q_{C}^{*}$, since the stress ratios are not mathematically derived, but chosen based on the judgement of the user. Determining $Q_{C}^{*}$ by optimizing the correlation of data on a Wilshire plot eliminates concern over the variance of $Q_{C}^{*}$ at arbitrarily-chosen stress ratios. The technique used to optimize the correlation of data is generally not defined in the literature and may vary between authors. In this work, $Q_{C}^{*}$ values ranging from 1 to 500 kJ/mol were iterated with a step size of 1 kJ/mol to find the best correlation, which was quantified by the coefficient of determination (R^2^).

Third, in some articles [5,24,25,26,27,28,29,32,36] the reasonableness of the calculated value of $Q_{C}^{*}$ is assessed by comparing it to an experimentally-measured or theoretically-calculated activation energy of lattice self-diffusion. If a value for the activation energy of lattice self-diffusion is known, it may be expedient to use this value as $Q_{C}^{*}$ rather than calculate a value from the experimental data.

After $Q_{C}^{*}$ has been determined, the *u* and *k*_1_ fitting constants are respectively defined as the slope and exponential of the *y*-intercept of a best fit line on a Wilshire plot. Multiple linear regions may be visible on this plot, which would suggest that the data be separated into regions to improve the goodness of fit of the Wilshire equation. Gray and Whittaker [37] point out that Wilshire split regions using two different methods. Regions were consistently split where *σ* was equal to *σ_YS_*, but in one case, $Q_{C}^{*}$ and new fitting constants were recalculated for each region [33], while in another case, the original $Q_{C}^{*}$ value was used and only the fitting constants were recalculated for each region [4]. The latter case is known to under-predict creep life [38], and the former case, which more accurately describes the underlying physical processes [37,39,40], was used by Whittaker and Wilshire to extrapolate the creep life of Grade 22, 23, and 24 steels [39]. In this study, both region-splitting techniques are used. Evans proposed a method to handle data from multiple batches [41], but this method requires a more complicated analysis that is outside the scope of this study, so it was not utilized.

### 3.3. Larson–Miller Parameter Equation

In the Larson–Miller parameter equation, the material constant *C* is often set to 20 [12]. However, *C* can be calculated if desired. The method described by Zhu et al. [42] was used to calculate *C* in this work. The accuracy of Equation (8), the LMP fitting function, increases with the number of terms that are used. For this study, four terms were deemed to be sufficient so the parameters *B*_0_, *B*_1_, *B*_2_ and *B*_3_ were obtained. With this information, *t_r_* can be estimated for any given combination of temperature and stress.

Following the method detailed by Zhu et al. [42], the matrix laboratory software (MATLAB, version 2018b) surface fitting tool was used to determine the LMP equation constants. The Larson–Miller parameter equation was arranged as:

(9) $$z=\frac{B_{0}+B_{1}y+B_{2}y^{2}+B_{3}y^{3}}{x}-C,$$

where

$$z=\log\left(t_{r}\right),\;\;x=T,\;\;y=\log\left(\sigma \right).$$

## 4. Results and Discussion

### 4.1. Investigation of Multiple Methods to Determine $Q_{C}^{*}$, Region-Splitting, the Use of Heat- and Processing-Specific Tensile Strength Values, and the Use of Short-Term Creep Rupture Data to Extrapolate Creep Life

**Figure 1:**
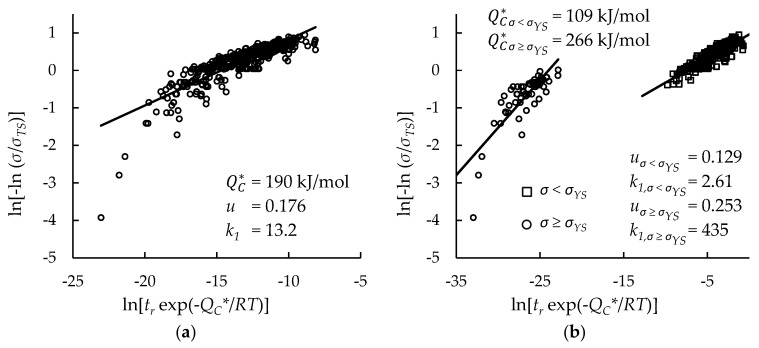

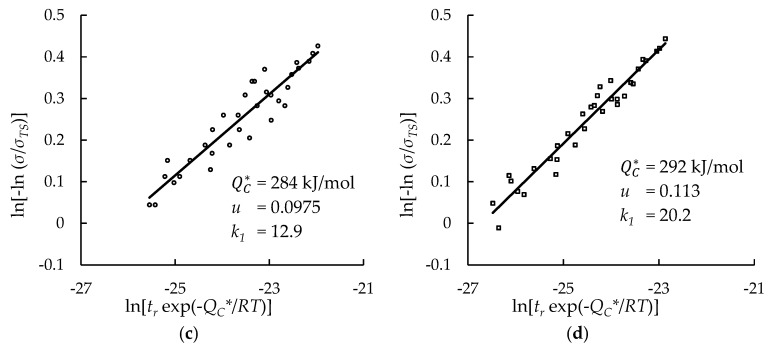
Goodness of fit of creep rupture data on Wilshire plots for: (**a**) Inconel 617 data treated as a single region; (**b**) Inconel 617 data split into above- and below-yield stress regions; (**c**) Nimonic 105 data with averaged tensile strength values; and (**d**) Nimonic 105 data with heat- and processing-specific tensile strength values.

**Figure A1:**
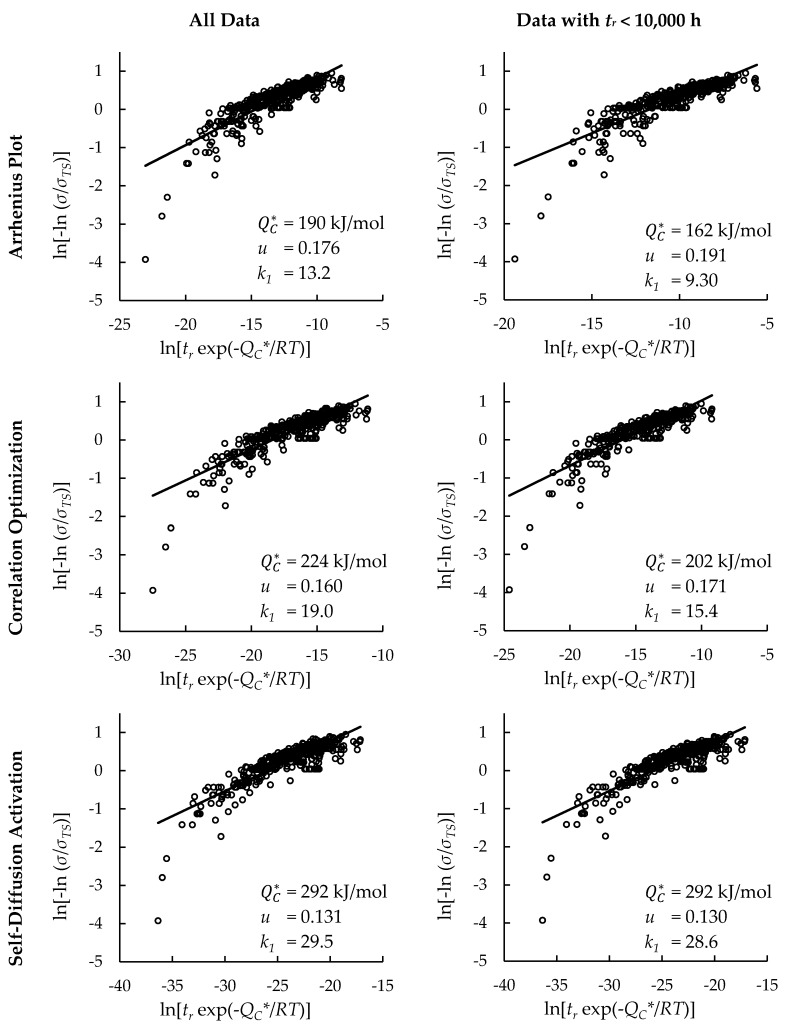
Wilshire plots of Inconel 617 creep rupture data treated as a single region.

**Figure A2:**
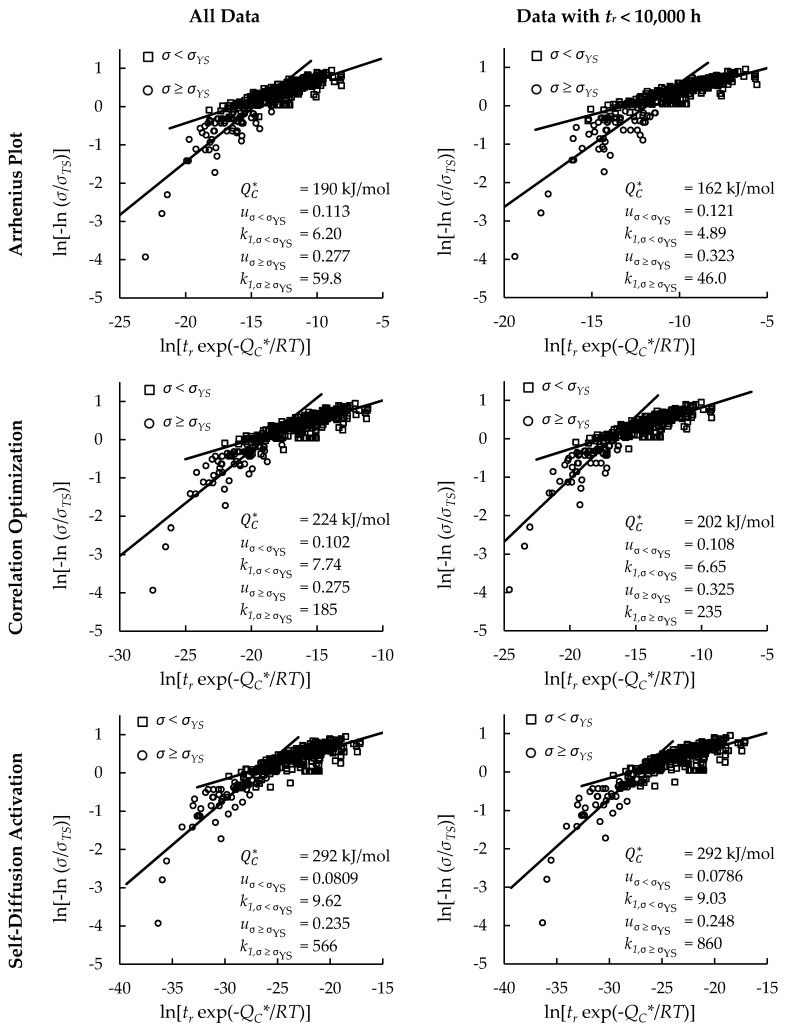
Wilshire plots of Inconel 617 creep rupture data split into above- and below-yield stress regions.

**Figure A3:**
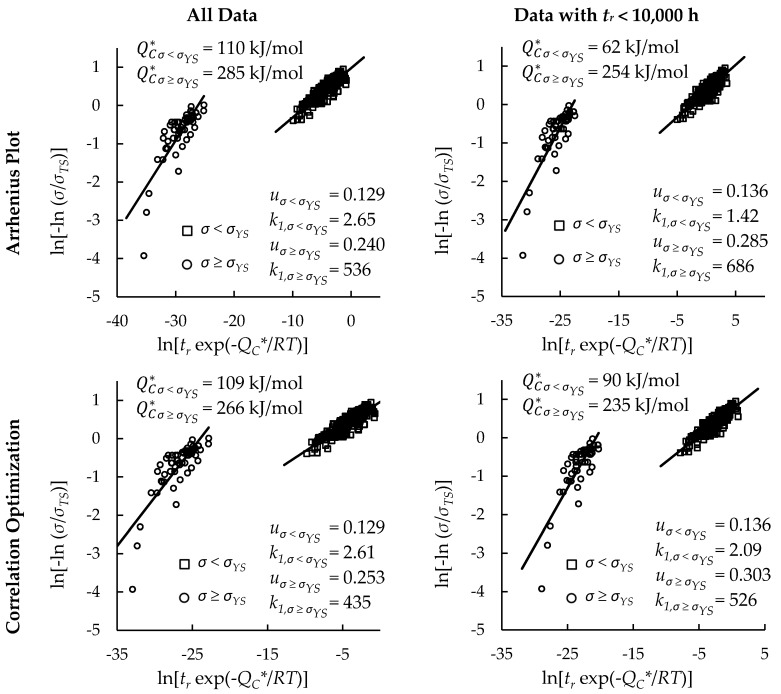
Wilshire plots of Inconel 617 creep rupture data split into above- and below-yield stress regions.

**Figure A4:**
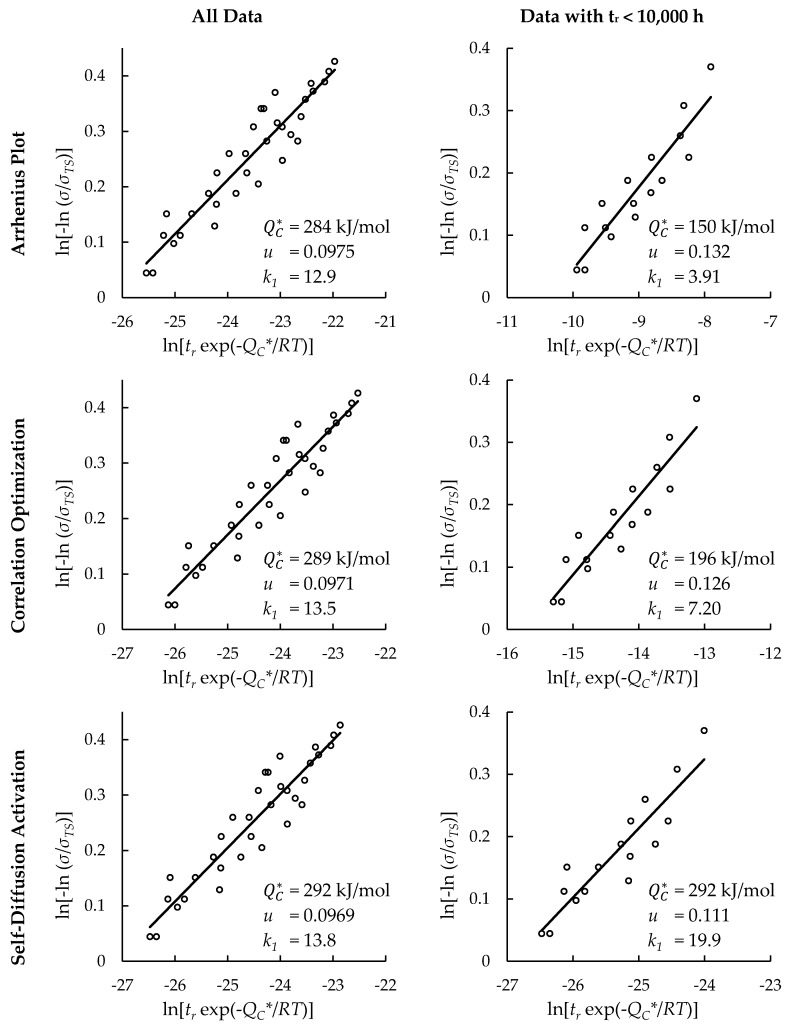
Wilshire plots of Nimonic 105 creep rupture data using average tensile strength values.

**Figure A5:**
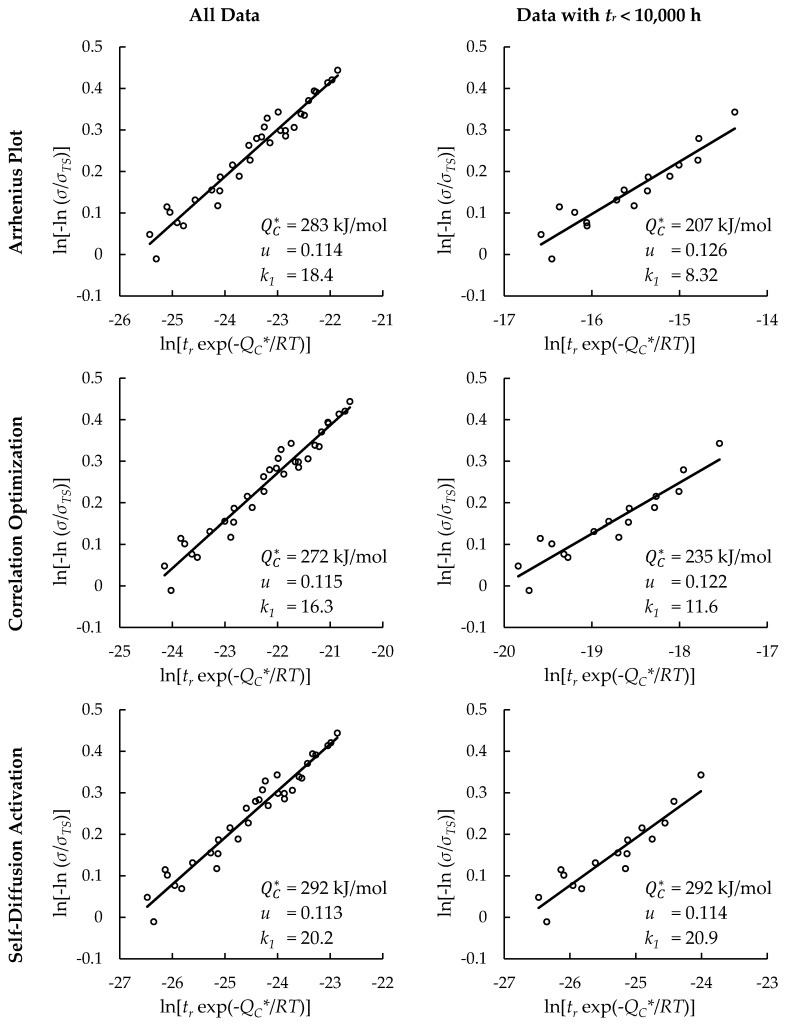
Wilshire plots of Nimonic 105 creep rupture data using heat- and processing-specific tensile strength values.

Creep rupture data for Inconel 617 and Nimonic 105 were split into two data sets for each alloy; one consisted of all data, while the other was limited to data with rupture times less than 10,000 h. The purpose of the limited data set is to show the efficacy of extrapolating short-term test data to longer times, as it has been claimed that the Wilshire equation is well-suited to do so [4,5,25,27,28,33]. For each data set, values of $Q_{C}^{*}$ were determined using Arrhenius plots (shown in Table 1 and Table 2), by optimizing the correlation of data on Wilshire plots (shown in Table 3 and Table 4), and using the activation energy of self-diffusion of nickel in a nickel lattice, 292 kJ/mol [43]. Since tensile strength values for each heat and processing condition are available for Nimonic 105, the effect of their use compared to the use of average values was investigated. Average tensile strength values were determined at each temperature using tensile strength values of all heats and processing conditions. Wilshire plots for each case were generated to show the goodness of fit of the data. To improve the goodness of fit, Wilshire plots for Inconel 617 were split into two regions: *σ* < *σ_YS_* and *σ* ≥ *σ_YS_*. Wilshire plots for Nimonic 105 were not split into regions because no data points have an applied stress higher than yield strength and no visible break appears in the data. For Inconel 617, negative $Q_{C}^{*}$ values were calculated using Arrhenius plots at two stress ratios, 0.4 and 0.5, for data with an applied stress less than yield strength. These negative $Q_{C}^{*}$ values were omitted from average $Q_{C}^{*}$ calculations. Representative Wilshire plots are shown in Figure 1. Plots of all cases are provided as Figure A1, Figure A2, Figure A3, Figure A4 and Figure A5 in Appendix A. For both alloys, $Q_{C}^{*}$ values much lower than the activation energy of self-diffusion of nickel in a nickel lattice were occasionally obtained. Similarly, low $Q_{C}^{*}$ values have been obtained by others [33,35,44].

For all cases, time to rupture was calculated at the stress and temperature of each experimental data point. The error of the calculated rupture times in hours was measured using mean squared error (MSE), defined as

(10) $$\mathrm{MSE}=\frac{\sum_{i=1}^{n}{\left(t_{r,calculated,i}-t_{r,experimental,i}\right)}^{2}}{n}.$$

The Wilshire equation’s goodness of fit (quantified by R^2^) for each data set and error obtained by applying the calculated $Q_{C}^{*}$ values and fitting constants to all creep rupture data are presented in Table 5 and Table 6.

For Inconel 617, the activation energy of self-diffusion of nickel in a nickel lattice gave the worst goodness of fit and error in all cases. Creep activation energy values at stresses below yield strength are much lower than those above yield strength. Both methods calculated much lower $Q_{C}^{*}$ values, ranging from 62 to 110 kJ/mol, than the published activation energy of self-diffusion of nickel in a nickel lattice, 292 kJ/mol. For data with an applied stress above yield strength, the calculated $Q_{C}^{*}$ values are slightly lower, but still near the activation energy of self-diffusion of nickel in a nickel lattice. Contrary to expectations, region splitting worsened the error in all cases. Additionally, using data with rupture times less than 10,000 h to calculate $Q_{C}^{*}$ and the fitting constants yielded errors similar to using all data. Regardless of the method used to calculate $Q_{C}^{*}$, relatively poor fits of the Wilshire equation to the Inconel 617 data were obtained. A similar issue of large data scatter of Inconel 617 has been reported by others [45,46].

For Nimonic 105, the goodness of fit and error improved dramatically from the use of heat- and processing-specific tensile strength values; for the data set with all data, the coefficient of determination increased from 0.88 to 0.95 and the mean squared error was reduced by about 70%. All three methods to determine $Q_{C}^{*}$ gave a similar goodness of fit and error with all data. Compared to using all data, the calculated $Q_{C}^{*}$ values are much lower and the goodness of fit is worse when using data with rupture times less than 10,000 h.

**Figure 2:**
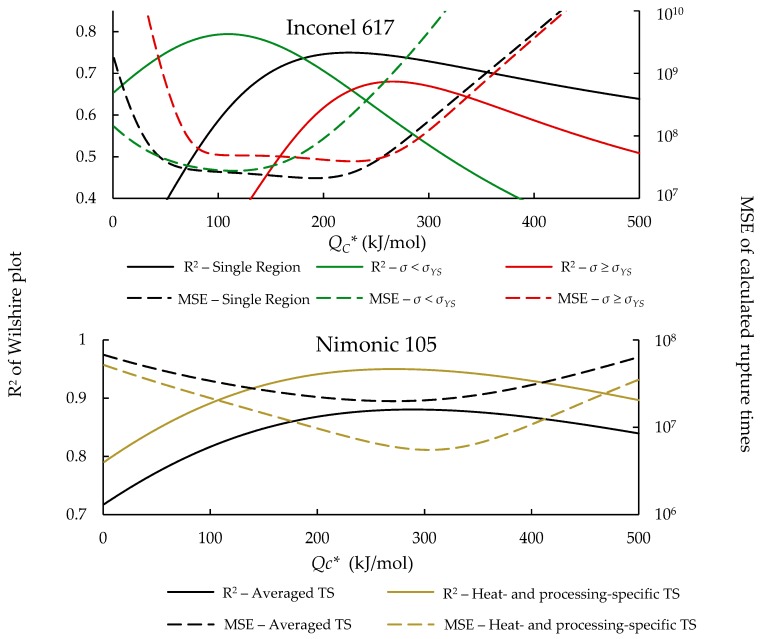
R^2^ and MSE vs. $Q_{C}^{*}$ for all data.

Figure 2 shows the correlation of the Wilshire equation and error at each potential value of $Q_{C}^{*}$, which were obtained when using the correlation optimization method to determine $Q_{C}^{*}$.

**Figure 3:**
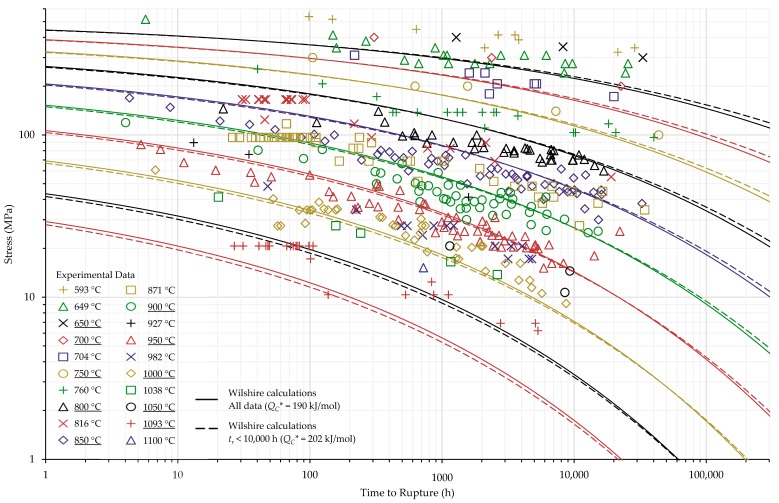
Single region calculations of the Wilshire equation with the lowest error for Inconel 617 shown at underlined temperatures.

**Figure 4:**
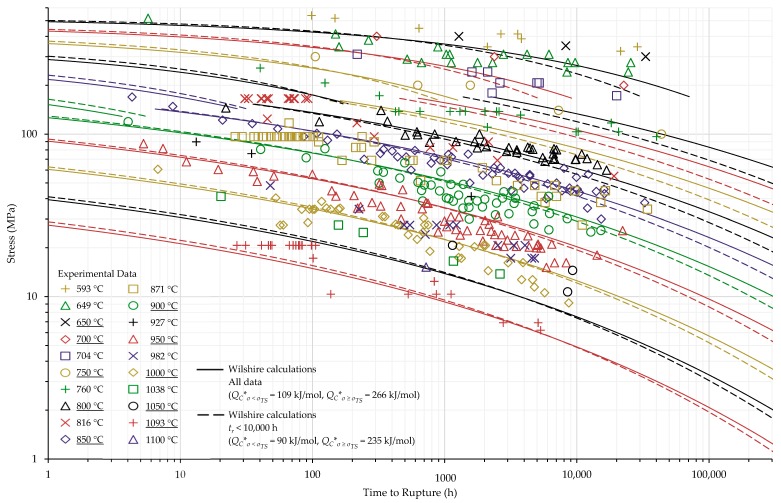
Split region calculations of the Wilshire equation with the lowest error for Inconel 617 shown at underlined temperatures.

**Figure 5:**
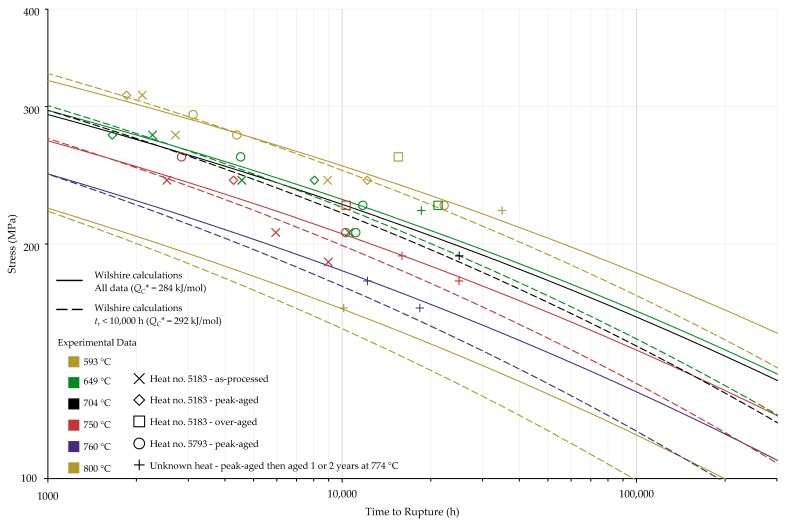
Calculations of the Wilshire equation with the lowest error for Nimonic 105 using averaged tensile strength values.

**Figure 6:**
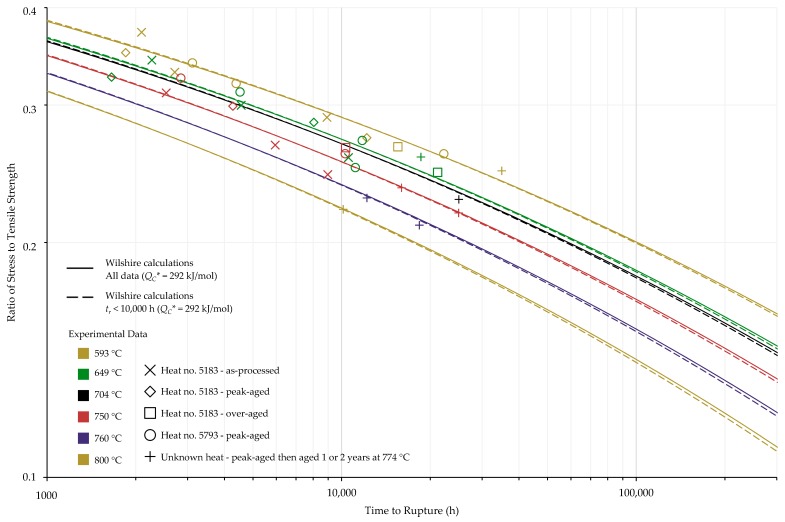
Calculations of the Wilshire equation with the lowest error for Nimonic 105 using heat- and processing-specific tensile strength values.

**Figure A6:**
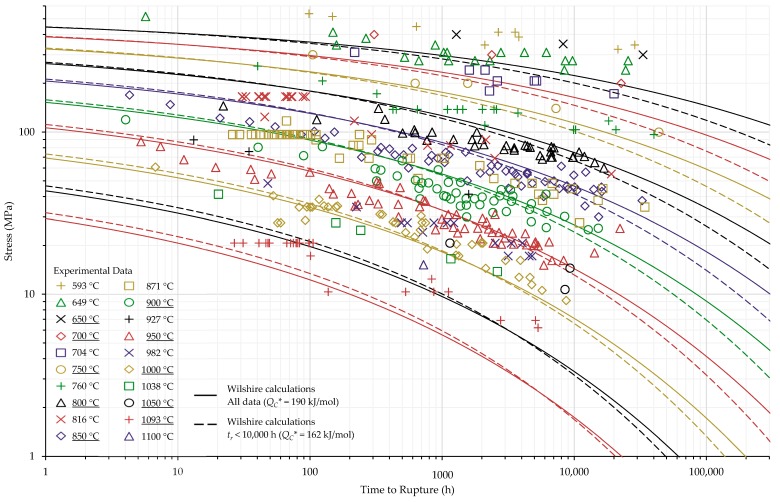
Single region calculations of the Wilshire equation with $Q_{C}^{*}$ determined using Arrhenius plots for Inconel 617 shown at underlined temperatures.

**Figure A7:**
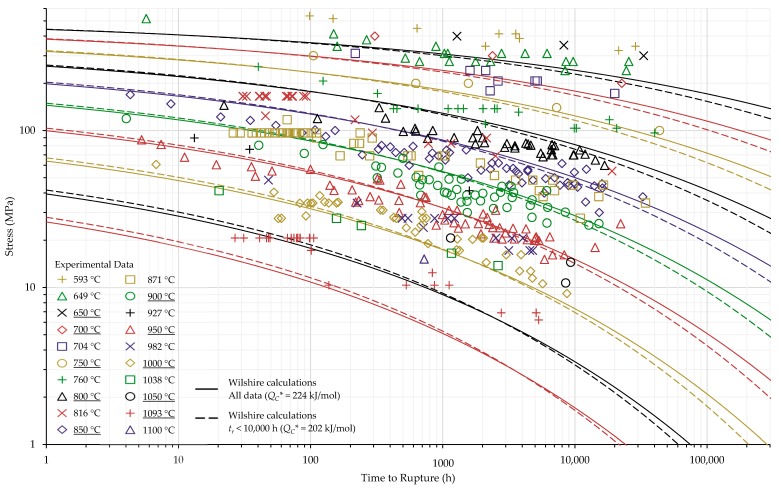
Single region calculations of the Wilshire equation with $Q_{C}^{*}$ determined by optimizing the correlation of data on a Wilshire plot for Inconel 617 shown at underlined temperatures.

**Figure A8:**
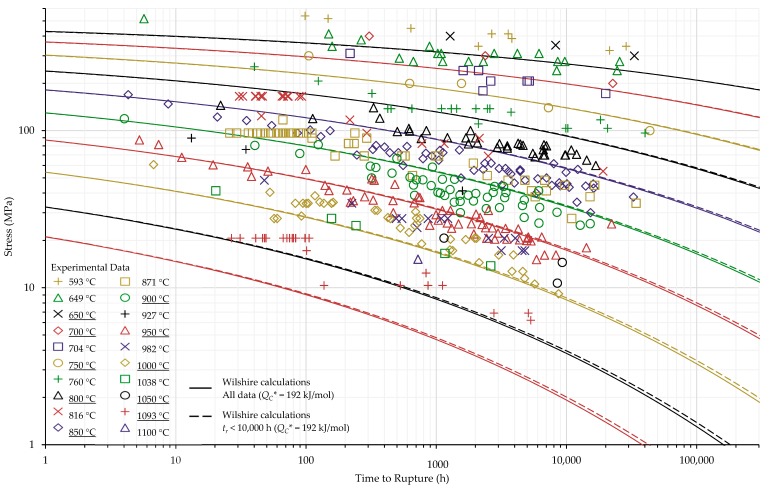
Single region calculations of the Wilshire equation with the self-diffusion activation energy of nickel in a nickel lattice as $Q_{C}^{*}$ for Inconel 617 shown at underlined temperatures.

**Figure A9:**
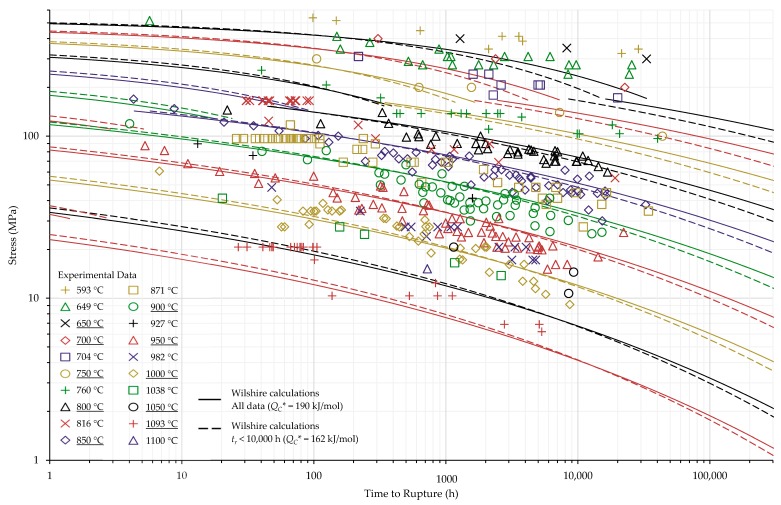
Split region calculations of the Wilshire equation with $Q_{C}^{*}$ determined using Arrhenius plots for Inconel 617 shown at underlined temperatures.

**Figure A10:**
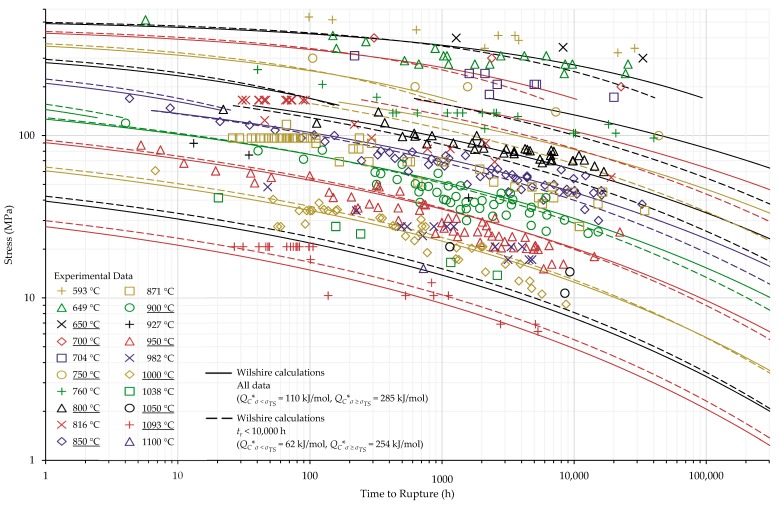
Split region calculations of the Wilshire equation with $Q_{C}^{*}$ determined using Arrhenius plots for Inconel 617 shown at underlined temperatures.

**Figure A11:**
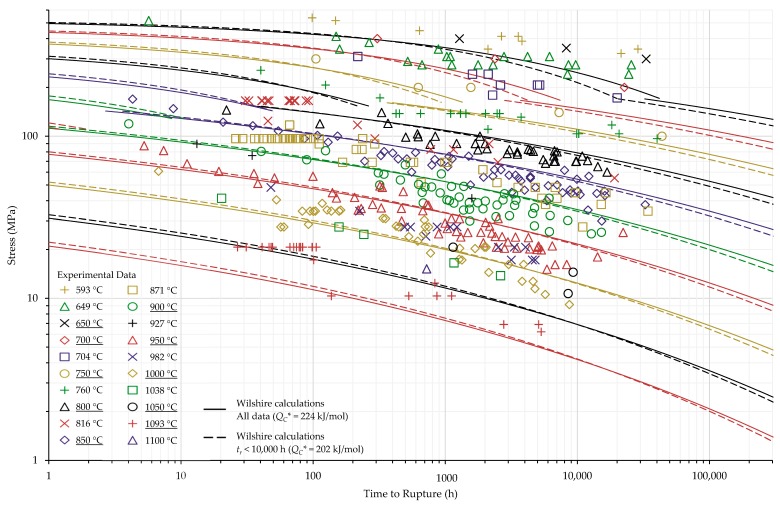
Split region calculations of the Wilshire equation with $Q_{C}^{*}$ determined by optimizing the correlation of data on a Wilshire plot for Inconel 617 shown at underlined temperatures.

**Figure A12:**
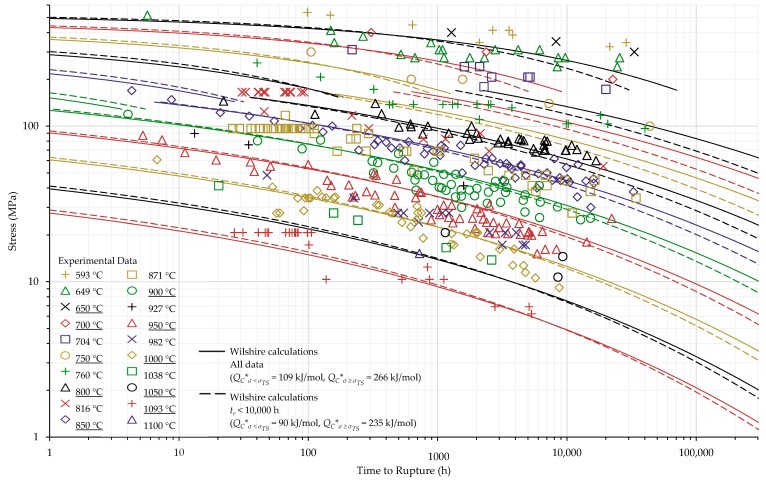
Split region calculations of the Wilshire equation with $Q_{C}^{*}$ determined by optimizing the correlation of data on a Wilshire plot for Inconel 617 shown at underlined temperatures.

**Figure A13:**
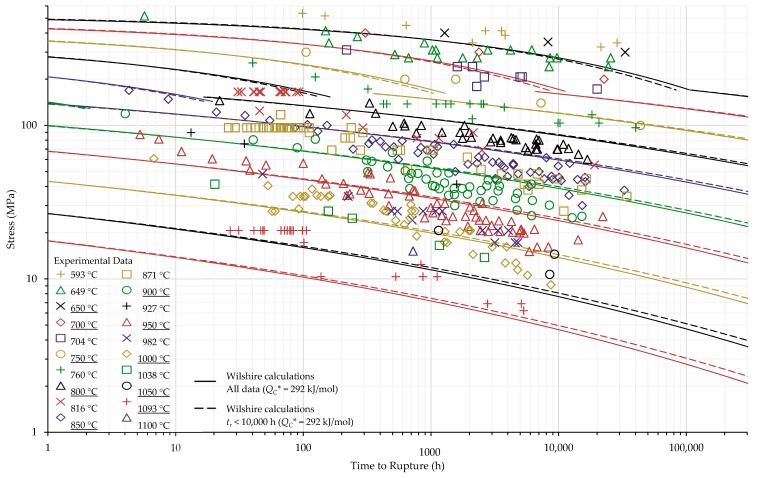
Single region calculations of the Wilshire equation with the self-diffusion activation energy of nickel in a nickel lattice as $Q_{C}^{*}$ for Inconel 617 shown at underlined temperatures.

**Figure A14:**
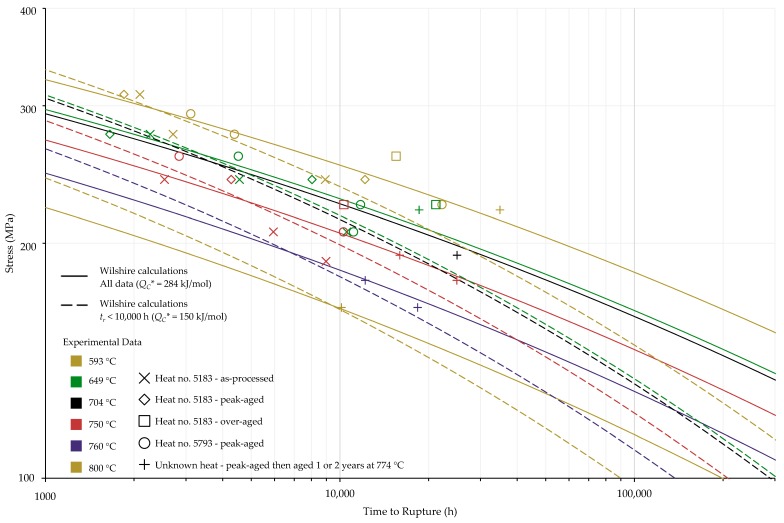
Calculations of the Wilshire equation for Nimonic 105 using average tensile strength values with $Q_{C}^{*}$ determined using Arrhenius plots.

**Figure A15:**
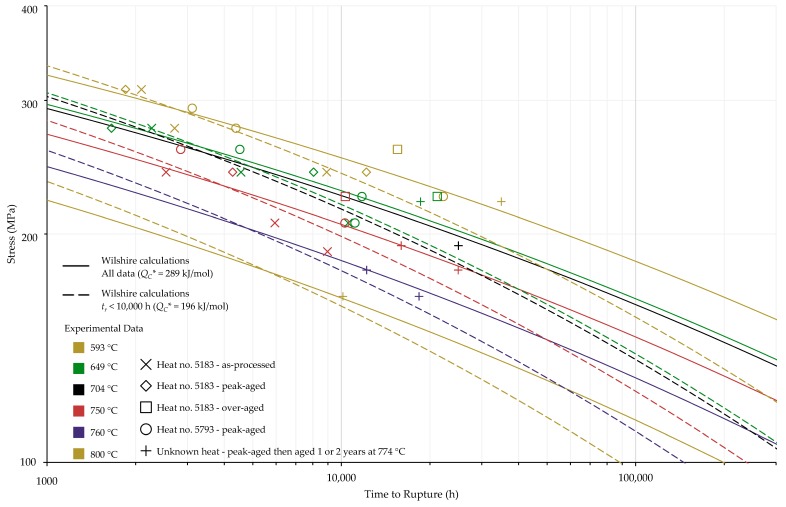
Calculations of the Wilshire equation for Nimonic 105 using average tensile strength values with $Q_{C}^{*}$ determined by optimizing the correlation of data on a Wilshire plot.

**Figure A16:**
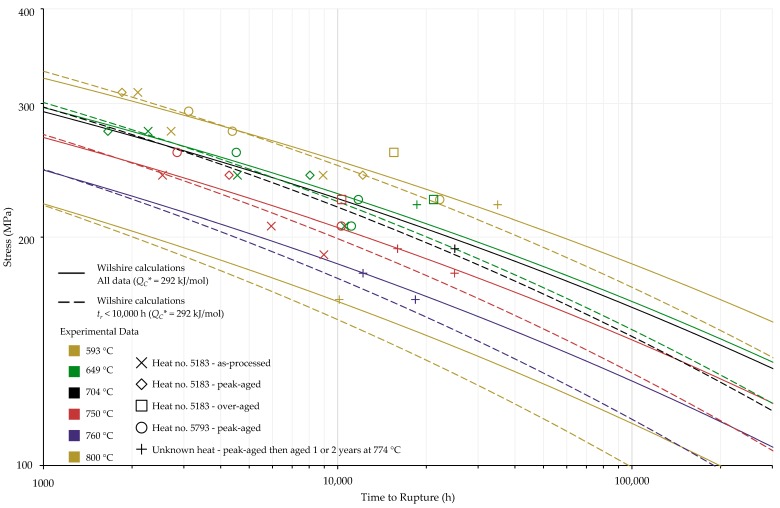
Calculations of the Wilshire equation for Nimonic 105 using average tensile strength values with the self-diffusion activation energy of nickel in a nickel lattice as $Q_{C}^{*}$.

**Figure A17:**
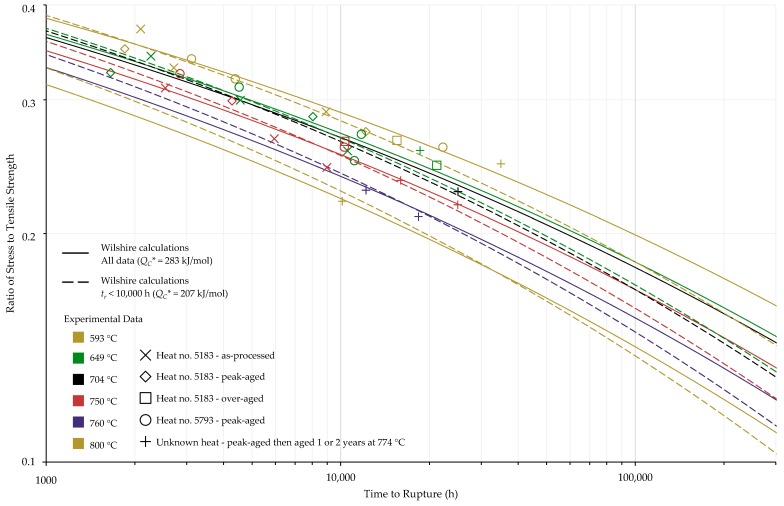
Split region calculations of the Wilshire equation for Nimonic 105 using heat- and processing-specific tensile strength values with $Q_{C}^{*}$ determined using Arrhenius plots.

**Figure A18:**
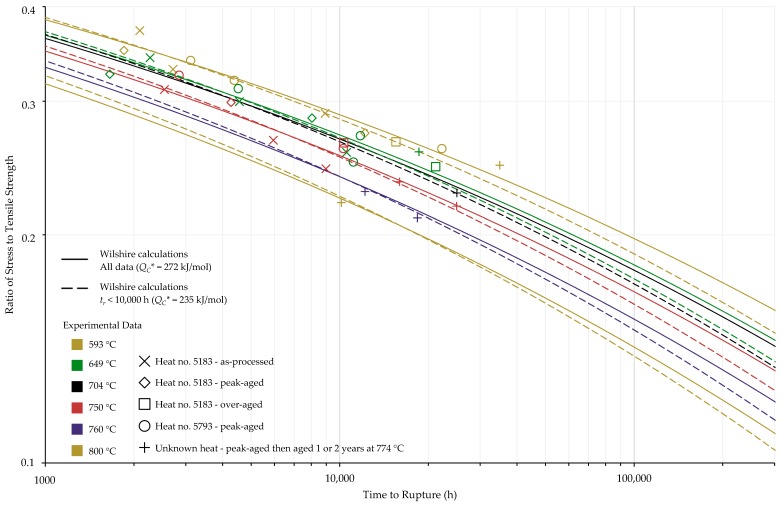
Calculations of the Wilshire equation for Nimonic 105 using heat- and processing-specific tensile strength values with $Q_{C}^{*}$ determined by optimizing the correlation of data on a Wilshire plot.

**Figure A19:**
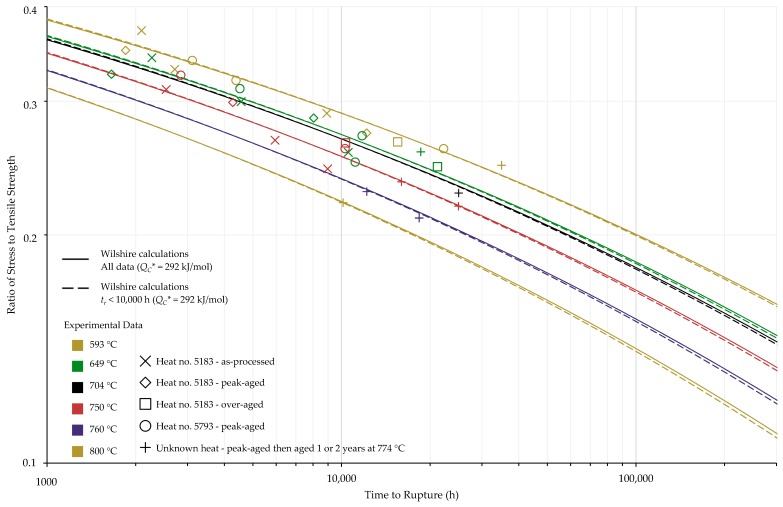
Calculations of the Wilshire equation for Nimonic 105 using heat- and processing-specific tensile strength values with the self-diffusion activation energy of nickel in a nickel lattice as $Q_{C}^{*}$.

The calculations of the Wilshire equation were plotted as stress vs. time to rupture. Plots for the method that provided the lowest error for each case are shown in Figure 3, Figure 4, Figure 5 and Figure 6, while the remaining plots are shown in Figure A6, Figure A7, Figure A8, Figure A9, Figure A10, Figure A11, Figure A12, Figure A13, Figure A14, Figure A15, Figure A16, Figure A17, Figure A18 and Figure A19 in Appendix A. For ease of displaying calculations for Nimonic 105 using heat- and processing-specific tensile strength values, the *y* axis of Figure 6 is shown as stress normalized by tensile strength. Calculated times to rupture for each data point and method to determine $Q_{C}^{*}$ for Nimonic 105 are shown in Table A2 in Appendix A. When splitting the data into above- and below-yield stress regions, the time to rupture at the transition from one region to the other is not calculated to be the same value in each region. Due to this, the split-region calculations of the Wilshire equation can yield zero or two stress values at some rupture times. The Inconel 617 plots show the tendency of the single-region rupture stress calculations to become more conservative than the split-region calculations as time increases.

From an engineering perspective, determining the average percentage difference between the calculated and experimentally-obtained rupture times is a reasonable way to show the tendency of the Wilshire equation to over- or under-predict creep life. For use by boiler pressure vessel design code organizations it is desirable that conservative estimations of creep life are produced. Average percentage difference is defined as

(11) $$\mathrm{Average}\;\mathrm{Percentage}\;\mathrm{Difference}=\frac{\sum_{i=1}^{n}\left(\frac{t_{r,calculated,i}-t_{r,experimental,i}}{t_{r,experimental,i}}\right)}{n}\times 100.$$

**Figure 7:**
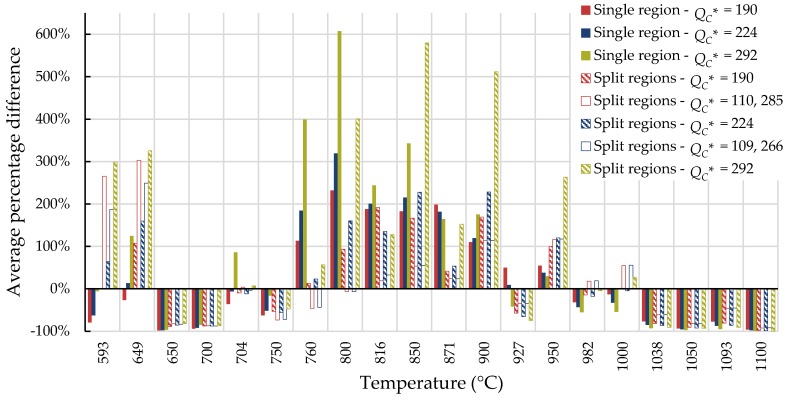
Average percentage difference at each temperature for Inconel 617 using all data to calculate $Q_{C}^{*}$ (kJ/mol).

**Figure 8:**
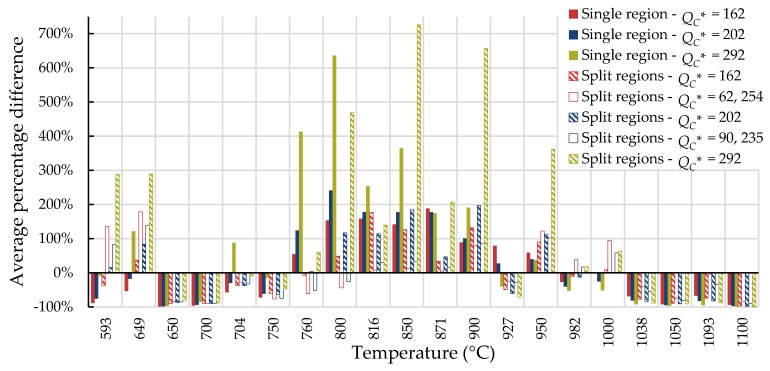
Average percentage difference at each temperature for Inconel 617 using data with times to rupture less than 10,000 h to calculate $Q_{C}^{*}$ (kJ/mol).

**Figure 9:**
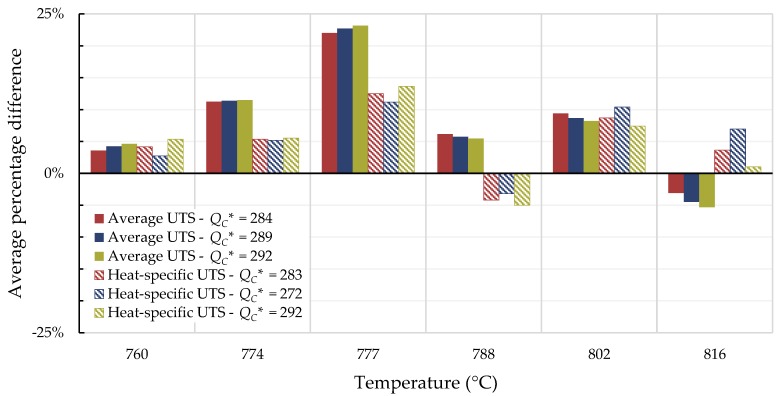
Average percentage difference at each temperature for Nimonic 105 using all data to calculate $Q_{C}^{*}$ (kJ/mol).

**Figure 10:**
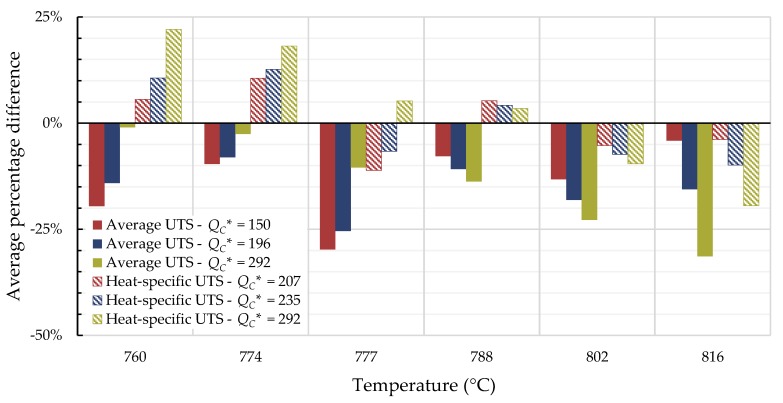
Average percentage difference at each temperature for Nimonic 105 using data with times to rupture less than 10,000 h to calculate $Q_{C}^{*}$ (kJ/mol).

For all cases, the average percentage difference was calculated at each temperature, and the results are shown in Figure 7, Figure 8, Figure 9 and Figure 10.

For Inconel 617, calculations from 800 to 900 °C are generally not conservative and those beyond 1000 °C tend to be conservative regardless of the method used. Use of the self-diffusion activation energy of nickel in a nickel lattice as $Q_{C}^{*}$ gave the largest overpredictions of creep life for single- and split-region analyses. For Nimonic 105, the use of heat- and processing-specific tensile strength data usually—but not always—improved the average percentage difference.

The European Creep Collaborative Committee (ECCC) has extrapolated the creep life of various alloys to 100,000 h, including Inconel 617 [47]. As mentioned by Bullough, et al. [48], an interim Inconel 617B ECCC data sheet exists and a revision is in progress. A comparison of the creep strengths for rupture at 100,000 h specified by the ECCC and those obtained using the Wilshire equation are shown in Table 7 at temperatures that are common to both the ECCC’s data sheet and the data used in this paper. The calculations of the Wilshire equation are closest to the values in the Inconel 617 ECCC data sheet when all data are treated as a single region and $Q_{C}^{*}$ is determined by optimizing the correlation of data on a Wilshire plot. Calculated creep strength values for rupture at 100,000 h for Nimonic 105 are presented in Table 8. For both alloys, the use of data with rupture times less than 10,000 h to calculate $Q_{C}^{*}$ generally resulted in lower calculated creep strengths for rupture at 100,000 h.

### 4.2. Comparison of Calculations of the Wilshire and Larson-Miller Parameter Equations

The Larson-Miller parameter equation was used to provide calculations for comparison with the calculations of the Wilshire equation. Equation (9) and the MATLAB surface fitting tool were used to correlate the experimental data to the LMP equation and the resulting coefficients and goodness of fit are shown in Table 9.

Time to rupture was calculated at the stress and temperature of each experimental data point using the LMP equation. The mean squared error of the calculated rupture times is compared to the lowest error obtained using the Wilshire equation with all data in Table 10. The error of the Wilshire calculations is lower than that of the LMP equation for both alloys. The best goodness of fit was achieved with the LMP equation for Inconel 617 and with the Wilshire equation for Nimonic 105.

**Figure 11:**
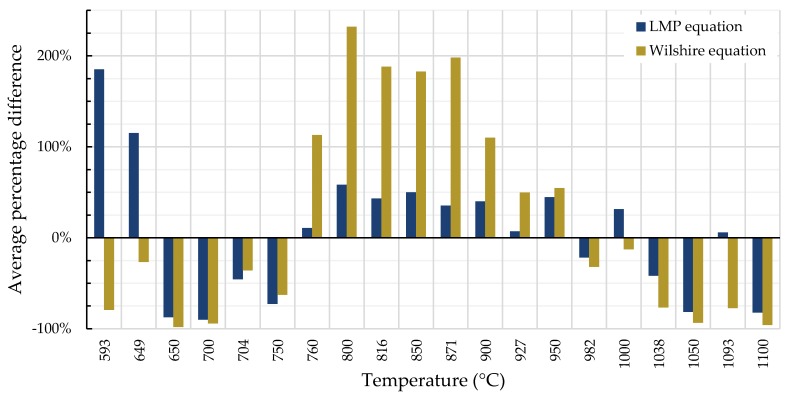
Average percentage difference of calculated and experimental rupture times for Inconel 617.

**Figure 12:**
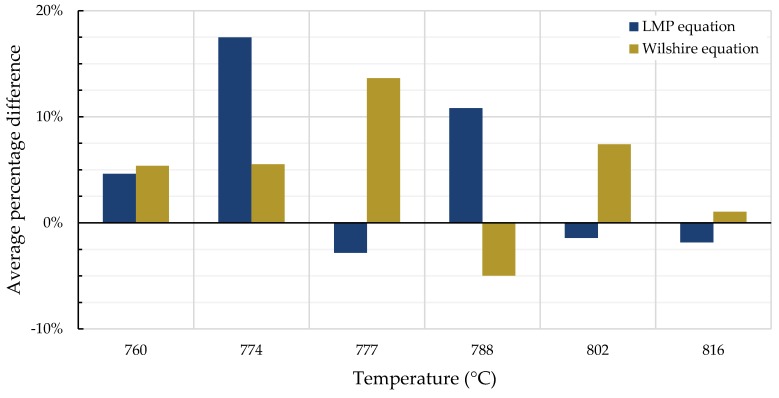
Average percentage difference of calculated and experimental rupture times for Nimonic 105.

A comparison of the tendency for each equation to over- or under-predict creep life—quantified as the average percentage difference between calculated and experimentally-obtained rupture times—is shown in Figure 11 and Figure 12. For Inconel 617, the over- and under-predictions of the LMP equation are generally less severe than those of the Wilshire equation, except at the two lowest temperatures.

The percentage differences of the calculated rupture time for the longest test duration of each alloy is defined as

(12) $$\mathrm{Percentage}\;\mathrm{Difference}=\frac{t_{r,calculated}-t_{r,experimental}}{t_{r,experimental}}\times 100.$$

As shown in Table 11 the calculated rupture time for both equations was conservative for each alloy compared to the longest experimental test data points. The LMP equation yielded more conservative estimates than the Wilshire equation.

**Figure 13:**
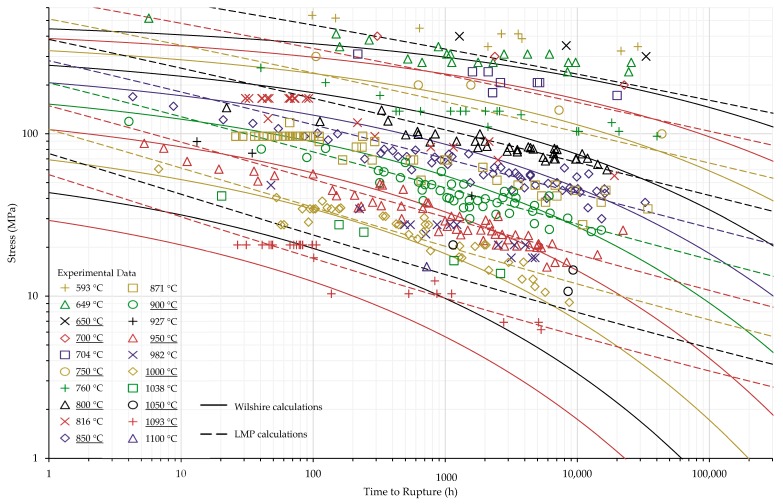
Calculated rupture times for Inconel 617 shown at underlined temperatures.

**Figure 14:**
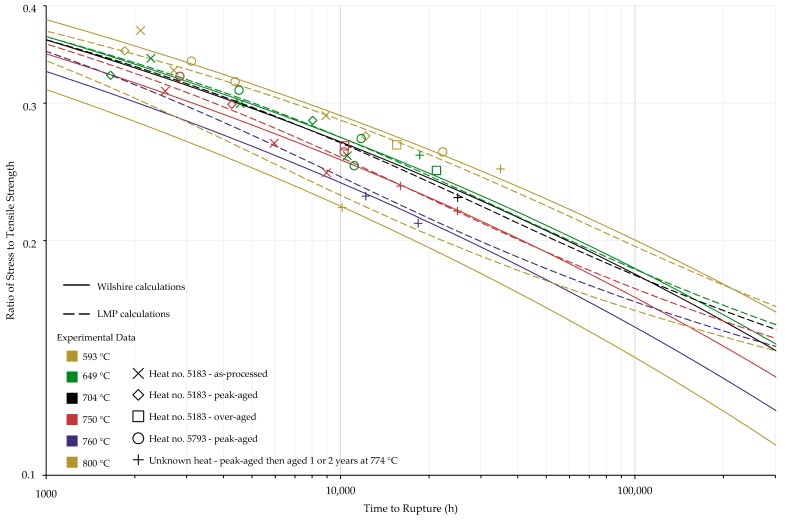
Calculated rupture times for Nimonic 105.

Calculated creep strengths for rupture at 100,000 h using the LMP and Wilshire equations are presented in Table 12 and Table 13. Figure 13 and Figure 14 show experimental creep data, calculations of the LMP equation, and calculations of the Wilshire equation using the $Q_{C}^{*}$ value that yielded the lowest error. For ease of displaying the calculations, the *y* axis of Figure 14 is stress normalized by tensile strength, and the stresses calculated using the LMP equation are normalized by average tensile strength values. Calculated times to rupture for each data point and method to determine $Q_{C}^{*}$ for Nimonic 105 are shown in Table A2 in Appendix A. The form of the stress function used in the LMP equation can result in multiple values of stress to be calculated at a single rupture time; however, this issue was not observed for either data set. For Inconel 617, the calculations of the Wilshire equation are more conservative than those of the LMP equation at failure times approaching and beyond 100,000 h. Nimonic 105 exhibited the same behavior at 800 and 850 °C. For Inconel 617, both the Wilshire and LMP equations predicted 100,000 h creep strengths lower or only slightly higher than the values calculated by the ECCC, including the temperature range of 800 to 900 °C where both equations overpredicted creep life vs. the experimental data (see Figure 11).

## 5. Conclusions

This study investigated multiple methods to determine $Q_{C}^{*}$, the effect of region-splitting, the use of short-term creep rupture data to extrapolate creep life, and the use of heat- and processing-specific tensile strength values, all of which are techniques that have been used or proposed to increase the accuracy of the Wilshire equation.

The large temperature span of Inconel 617 data, over 500 °C, may be the cause for relatively poor fits of both the Wilshire and LMP models to the data. At higher temperatures, greater than 850 °C, microstructural changes that affect creep strength may be accelerated, or other strength degradation phenomena might become significant that do not occur at lower temperatures. For Inconel 617, the mean squared error of the calculated creep life using the self-diffusion activation energy of nickel in a nickel lattice as $Q_{C}^{*}$ was about an order of magnitude greater than the other methods to determine $Q_{C}^{*}$. The large scatter and temperature range may have exacerbated potential error introduced by using the self-diffusion activation energy as $Q_{C}^{*}$, rather than using a value calculated from the data. With the well-behaved Nimonic 105 data, a very similar goodness of fit was obtained using any of the three methods for determining $Q_{C}^{*}$. It is possible that calculations of the Wilshire equation are not significantly affected by the $Q_{C}^{*}$ value when a small data set with low scatter is used. If a high degree of fit to the data is required, the authors recommend that self-diffusion activation energy not be used as $Q_{C}^{*}$ due to the potential for large error, as seen for Inconel 617. Using the $Q_{C}^{*}$ value that provided the lowest error, the longest time to rupture for both alloys was underpredicted, which is much more desirable than overprediction. Contrary to expectations, treating the data as a single region—rather than splitting the data into above- and below-yield stress regions—provided the lowest mean squared errors for Inconel 617. The use of heat- and processing-specific tensile strength values greatly improved the goodness of fit of the Wilshire equation to the Nimonic 105 data and reduced the error in all cases. For Inconel 617, the use of data with rupture times less than 10,000 h to extrapolate creep life gave roughly similar goodness of fit and error compared to using all data. Surprisingly, for Nimonic 105 the use of data with rupture times less than 10,000 h to extrapolate creep life resulted in a significant reduction in goodness of fit and error compared to using all data, e.g., R^2^ of 0.880 versus 0.950 when $Q_{C}^{*}$ was determined using the correlation optimization method. Considering that the longest Nimonic 105 experimental data point was 34,995 h time to rupture, this result would suggest that further investigation should be made of the ability of the Wilshire equation to accurately predict long-term creep strength from short-term creep rupture strength data.

In its basic form, the Wilshire equation is a simple method to quickly estimate long-term creep life using only three fitting constants—yet if an extensive analysis with a high level of precision is required, its complexity can be increased to improve the statistical fit of the Wilshire equation to available data. Evans [41,49,50,51,52,53,54] has proposed more sophisticated methods of fitting the Wilshire equation to complex data sets, including the handling of data collected from specimens of multiple batches [41], determining $Q_{C}^{*}$ as a function of temperature [41], statistically determining the number of stress regions [49], and utilizing additional batch characteristics [50]. The Wilshire equation is modular in that many combinations of these methods can be used, which gives it flexibility for a wide variety of applications. If only a preliminary estimate of long-term creep strength (e.g., at 100,000 h or longer design life) is needed, such as in the early stages of new alloy development, use of the Wilshire equation in its original form with $Q_{C}^{*}$ equal to the activation energy of self-diffusion would probably be sufficient. More complex analyses (which, in essence, increase the number of fitting constants) would be needed if the intent is to use the Wilshire equation for component design or for establishing long-term creep strength values for design codes, instead of the equations now used in various design codes, and which contain more than three fitting constants.

## Figures and Tables

**Table 1:** Inconel 617 $Q_{C}^{*}$ values (kJ/mol) determined using Arrhenius plots.

Data Set	Stress Region	Average $Q_{C}^{*}$	$\sigma /\sigma_{TS}$								
			0.1	0.2	0.3	0.4	0.5	0.6	0.7	0.8	0.9
All Data	All *σ*	190	63	122	118	193	252	362	173	28	401
	σ < σ_YS_	110	72	152	108	−12 ^a^	−154 ^a^	–	–	–	–
	σ ≥ σ_YS_	285	–	–	–	530	318	274	161	28	401
*t_r_* < 10,000 h	All *σ*	162	50	60	106	157	227	307	151	26	376
	σ < σ_YS_	62	45	68	73	−6 ^a^	−132 ^a^	–	–	–	–
	σ ≥ σ_YS_	254	–	–	–	413	285	278	148	26	376

^a^ Omitted from average $Q_{C}^{*}$ calculation.

**Table 2:** Nimonic 105 $Q_{C}^{*}$ values (kJ/mol) determined using Arrhenius plots.

Data Set	Tensile Strength	Average $Q_{C}^{*}$	$\sigma /\sigma_{TS}$	
			0.2	0.3
All Data	Average	284	372	196
	Heat- and processing-specific	283	311	255
*t_r_* < 10,000 h	Average	150	–	150
	Heat- and processing-specific	207	–	207

**Table 3:** Inconel 617 $Q_{C}^{*}$ values (kJ/mol) determined using Arrhenius plots.

Data Set	Tensile Strength	$Q_{C}^{*}$
All Data	All *σ*	224
	*σ* < *σ_YS_*	109
	*σ* ≥ *σ_YS_*	266
*t_r_* < 10,000 h	All *σ*	202
	*σ* < *σ_YS_*	90
	*σ* ≥ *σ_YS_*	235

**Table 4:** Nimonic 105 $Q_{C}^{*}$ values (kJ/mol) determined by optimizing the correlation of data on Wilshire plots.

Data Set	Tensile Strength	$Q_{C}^{*}$
All Data	Average	289
	Heat- and processing-specific	272
*t_r_* < 10,000 h	Average	196
	Heat- and processing-specific	235

**Table 5:** Quality of fit and error for Inconel 617.

Data Set	Split Regions?	$Q_{C}^{*}$ Determination Method	$Q_{C}^{*}\;\mathrm{All}\;\sigma$	$Q_{C}^{*}\;\sigma <\sigma_{\mathrm{YS}}$	$Q_{C}^{*}\;\sigma \geq \sigma_{\mathrm{YS}}$	R^2^ All *σ*	R^2^ *σ* *<* *σ_YS_*	R^2^ *σ* ≥ *σ_YS_*	MSE
All Data	No	Arrhenius Plot	190	—	—	0.742	—	—	$2.11\times 10^{7}$
		Correlation Optimization	224	—	—	0.750	—	—	$2.49\times 10^{7}$
		Self-Diffusion Activation	292	—	—	0.732	—	—	$1.35\times 10^{8}$
	Yes	Arrhenius Plot	190	—	—	—	0.722	0.594	$6.45\times 10^{7}$
			—	110	285	—	0.794	0.677	$3.58\times 10^{7}$
		Correlation Optimization	224	—	—	—	0.665	0.657	$1.74\times 10^{8}$
			—	109	266	—	0.794	0.680	$3.16\times 10^{7}$
		Self-Diffusion Activation	292	—	—	—	0.542	0.674	$2.74\times 10^{9}$
*t_r_* < 10,000 h	No	Arrhenius Plot	162	—	—	0.724	—	—	$2.31\times 10^{7}$
		Correlation Optimization	202	—	—	0.741	—	—	$2.10\times 10^{7}$
		Self-Diffusion Activation	292	—	—	0.720	—	—	$1.56\times 10^{8}$
	Yes	Arrhenius Plot	162	—	—	—	0.757	0.568	$3.80\times 10^{7}$
			—	62	254	—	0.795	0.701	$2.96\times 10^{7}$
		Correlation Optimization	202	—	—	—	0.691	0.671	$9.96\times 10^{7}$
			—	90	235	—	0.811	0.700	$2.69\times 10^{7}$
		Self-Diffusion Activation	292	—	—	—	0.530	0.682	$4.76\times 10^{9}$

**Table 6:** Quality of fit and error for Nimonic 105.

Data Set	Tensile Strength	$Q_{C}^{*}$ Determination Method	$Q_{C}^{*}$ All *σ*	R^2^ All *σ*	MSE
All Data	Average	Arrhenius Plot	284	0.880	$2.00\times 10^{7}$
		Correlation Optimization	289	0.880	$2.01\times 10^{7}$
		Self-Diffusion Activation	292	0.880	$2.02\times 10^{7}$
	Heat- and processing-specific	Arrhenius Plot	283	0.950	$5.71\times 10^{6}$
		Correlation Optimization	272	0.950	$5.96\times 10^{6}$
		Self-Diffusion Activation	292	0.949	$5.58\times 10^{6}$
*t_r_* < 10,000 h	Average	Arrhenius Plot	150	0.849	$3.24\times 10^{7}$
		Correlation Optimization	196	0.854	$2.86\times 10^{7}$
		Self-Diffusion Activation	292	0.838	$2.02\times 10^{7}$
	Heat- and processing-specific	Arrhenius Plot	207	0.879	$1.98\times 10^{7}$
		Correlation Optimization	235	0.880	$1.81\times 10^{7}$
		Self-Diffusion Activation	292	0.875	$1.67\times 10^{7}$

**Table 7:** Calculated creep strength for rupture of Inconel 617 at 100,000 h (MPa).

Data Set	Split Regions?	$Q_{C}^{*}\;\mathrm{Determination}\;\mathrm{Method}$	$Q_{C}^{*}\;\mathrm{All}\;\sigma$	$Q_{C}^{*}\;\sigma <\sigma_{\mathrm{YS}}$	$Q_{C}^{*}\;\sigma \geq \sigma_{\mathrm{YS}}$	650 (°C)	700 (°C)	750 (°C)	760 (°C)	800 (°C)	850 (°C)	900 (°C)
All Data	No	Arrhenius Plot	190	—	—	146	95.8	59.0	53.2	34.0	18.3	9.07
		Correlation Optimization	224	—	—	171	115	71.8	64.8	41.8	22.6	11.2
		Self-Diffusion Activation	292	—	—	210	146	94.5	86.0	57.1	32.0	16.4
	Yes	Arrhenius Plot	190	—	—	131	96.1	67.9	63.1	46.3	30.3	18.8
			—	110	285	83.6	63.3	46.6	43.8	33.4	23.2	15.3
		Correlation Optimization	224	—	—	148	109	77.2	71.8	52.7	34.4	21.3
			—	109	266	83.0	62.9	46.4	43.6	33.3	23.1	15.3
		Self-Diffusion Activation	292	—	—	173	128	92.5	86.3	64.2	42.7	26.8
*t_r_* < 10,000 h	No	Arrhenius Plot	162	—	—	118	76.2	46.2	41.5	26.3	14.0	6.93
		Correlation Optimization	202	—	—	153	101	62.4	56.2	35.9	19.2	9.44
		Self-Diffusion Activation	292	—	—	210	146	95.4	86.9	58.0	32.7	16.9
	Yes	Arrhenius Plot	162	—	—	114	83.9	59.5	55.3	40.7	26.9	16.8
			—	62	254	54.7	43.6	34.0	32.2	25.8	19.0	13.4
		Correlation Optimization	202	—	—	137	101	71.8	66.7	49.0	32.2	20.0
			—	90	235	69.1	52.8	39.4	37.0	28.6	20.1	13.5
		Self-Diffusion Activation	292	—	—	N/A ^1^	129	93.9	87.8	65.7	44.1	28.0
ECCC Inconel 617 Data Sheet (Year: 2005)						179	112	68	62	41	24	14.9
ECCC Interim Inconel 617B Data Sheet (Year: 2014)						222	129	70.6	62.7	39.9	—	—

^1^ The Wilshire equation did not yield a creep strength for rupture at 100,000 h in either the above- or below-yield stress region calculations (see Figure A13).

**Table 8:** Calculated creep strength for rupture of Nimonic 105 at 100,000 h (MPa).

Data Set	Tensile Strength	$Q_{C}^{*}\;\mathrm{Determination}\;\mathrm{Method}$	$Q_{C}^{*}$	Heat and Processing Condition	760 (°C)	774 (°C)	777 (°C)	788 (°C)	802 (°C)	816 (°C)
All Data	Average	Arrhenius Plot	284	—	183	164	161	146	129	114
		Correlation Optimization	289	—	184	164	161	146	129	114
		Self-Diffusion Activation	292	—	184	164	161	146	129	113
	Heat- and processing-specific	Arrhenius Plot	283	5183 AP	166	148	144	131	116	102
				5183 PA	176	155	151	136	119	103
				5183 OA	194	167	162	143	121	102
				5793 PA	172	152	148	134	118	103
				Unknown PA ^1^	178	157	153	139	122	106
		Correlation Optimization	272	5183 AP	165	147	143	130	115	102
				5183 PA	175	154	150	136	119	103
				5183 OA	193	166	161	142	121	102
				5793 PA	170	151	148	134	118	104
				Unknown PA ^1^	176	156	153	138	122	107
		Self-Diffusion Activation	292	5183 AP	167	148	145	131	116	102
				5183 PA	177	156	152	136	119	103
				5183 OA	195	168	163	143	121	102
				5793 PA	173	153	149	134	118	103
				Unknown PA ^1^	179	158	154	139	122	106
*t_r_* < 10,000 h	Average	Arrhenius Plot	150	—	148	134	132	121	109	97
		Correlation Optimization	196	—	155	139	136	124	110	97
		Self-diffusion Activation	292	—	172	151	148	132	115	99
	Heat- and processing-specific	Arrhenius Plot	207	5183 AP	153	138	135	124	111	99
				5183 PA	162	145	141	129	114	100
				5183 OA	179	156	151	135	116	99
				5793 PA	158	142	139	127	113	101
				Unknown PA ^1^	164	147	143	131	117	103
		Correlation Optimization	235	5183 AP	157	141	138	126	112	99
				5183 PA	167	148	144	131	115	101
				5183 OA	184	159	155	137	117	99
				5793 PA	163	145	142	129	114	101
				Unknown PA ^1^	168	150	146	133	118	104
		Self-Diffusion Activation	292	5183 AP	166	147	144	130	115	101
				5183 PA	177	155	151	136	118	102
				5183 OA	194	167	162	142	120	101
				5793 PA	172	152	148	134	117	102
				Unknown PA ^1^	178	157	153	138	121	105

^1^ Aged for 1–2 years at 774 °C.

**Table 9:** MATLAB calculations of the LMP coefficients and goodness of fit.

Alloy	$B_{0}$	$B_{1}$	$B_{2}$	$B_{3}$	*C*	R^2^
Inconel 617	32,630	−8114	1749	−357.2	16.02	0.837
Nimonic 105	354,200	−426,900	185,600	−27,280	16.88	0.842

**Table 10:** Goodness of fit and error of Wilshire and LMP calculations.

Alloy	Equation	R^2^	MSE
Inconel 617	LMP Equation	0.837	$2.69\times 10^{7}$
	Wilshire Equation ^1^	0.742	$2.11\times 10^{7}$
Nimonic 105	LMP Equation	0.842	$1.68\times 10^{7}$
	Wilshire Equation ^2^	0.949	$5.58\times 10^{6}$

^1^ Data treated as a single region with $Q_{C}^{*}$ calculated using Arrhenius plots; ^2^ Heat- and processing-specific tensile strength data with the self-diffusion activation energy of nickel in a nickel lattice as $Q_{C}^{*}$.

**Table 11:** Percentage differences of the calculated rupture times for the longest test durations.

Alloy	Calculation Method	Percentage Difference	Experimental Values of Longest Test Duration		
			Temperature (°C)	Stress (MPa)	Time to Rupture (h)
Inconel 617	LMP Equation	−73.8%	750	100	43,706
	Wilshire Equation ^1^	−61.0%			
Nimonic 105	LMP Equation	−31.2%	760	221	34,955
	Wilshire Equation ^2^	−17.2%			

^1^ Data treated as a single region with $Q_{C}^{*}$ calculated using Arrhenius plots; ^2^ Heat- and processing-specific tensile strength data with the self-diffusion activation energy of nickel in a nickel lattice as $Q_{C}^{*}$.

**Table 12:** Calculated creep strength for rupture at 100,000 h (MPa) of Inconel 617.

Calculation Method	650 °C	700 °C	750 °C	800 °C	850 °C	900 °C	950 °C	1000 °C
LMP Equation	161	104	66.0	41.7	26.4	16.8	10.8	7.13
Wilshire Equation ^1^	145	95.8	59.0	34.0	18.3	9.07	4.14	1.72
ECCC (Year: 2005)	179	112	68	41	24	14.9	—	—

^1^ Data treated as a single region with $Q_{C}^{*}$ calculated using Arrhenius plots.

**Table 13:** Calculated creep strength for rupture at 100,000 h (MPa) of Nimonic 105.

Calculation Method	Heat and Processing Condition	700 °C	750 °C	800 °C	850 °C
LMP Equation	—	273	189	129	99.7
Wilshire Equation^1^	5183 AP	270	182	117	72.5
	5183 PA	297	194	121	70.8
	5183 OA	351	217	124	63.1
	5793 PA	282	188	120	72.7
	Unknown PA ^2^	294	195	124	74.4

^1^ Heat- and processing-specific tensile strength data with the self-diffusion activation energy of nickel in a nickel lattice as $Q_{C}^{*}$; ^2^ Aged for 1–2 years at 774 °C.

## Disclaimer

This report was prepared as an account of work sponsored by an agency of the United States Government. Neither the United States Government nor any agency thereof, nor any of their employees, makes any warranty, express or implied, or assumes any legal liability or responsibility for the accuracy, completeness, or usefulness of any information, apparatus, product, or process disclosed, or represents that its use would not infringe privately owned rights. Reference therein to any specific commercial product, process, or service by trade name, trademark, manufacturer, or otherwise does not necessarily constitute or imply its endorsement, recommendation, or favoring by the United States Government or any agency thereof. The views and opinions of authors expressed therein do not necessarily state or reflect those of the United States Government or any agency thereof.

## Appendix A

**Table A1:** Nimonic 105 tensile strength values [3,4,21].

Temperature	Heat No.	Processing Condition	Tensile Strength (MPa)	Yield Strength (MPa)
760 °C	5183	AP	834	737
		PA	886	724
		OA	975	722
	5793	PA	863	719
	Unknown	PA ^1^	893	687
816 °C	5183	AP	718	680
		PA	728	625
		OA	720	605
	5793	PA	729	672
	Unknown	PA ^1^	751	610

^1^ Aged for 1–2 years at 774 °C.

**Table A2:** Calculated times to rupture (h) for each method to determine $Q_{C}^{*}$ for Nimonic 105.

Heat No. [3,4,21]	Processing Condition [3,4,21]	Temperature (°C) [3,4,21]	Applied Stress (MPa) [3,4,21]	Heat- and Processing-Specific TS (MPa) [3,4,21]	Experimental Time to Rupture (h) [3,4,21]	Calculated Time to Rupture (h)												
						Case 1	Case 2	Case 3	Case 4	Case 5	Case 6	Case 7	Case 8	Case 9	Case 10	Case 11	Case 12	LMP Equation
5183	AP	760	310	834	2092	1545	1545	1545	1771	1773	1768	1281	1324	1340	1401	1400	1378	1729
5183	AP	760	276	834	2713	4617	4638	4651	4572	4514	4620	3170	3350	3637	5054	5263	5717	4950
5183	AP	760	241	834	8914	14,068	14,201	14,281	12,011	11,688	12,284	7934	8571	9998	11,949	12,795	14,843	13,395
5183	AP	774	276	805	2268	1999	1989	1984	2091	2110	2075	1933	1891	1734	2699	2665	2589	2640
5183	AP	774	241	805	4560	6351	6352	6352	5695	5663	5722	4979	4982	4920	6590	6697	6966	7052
5183	AP	774	207	805	10,546	20,879	20,988	21,055	15,976	15,643	16,261	13,132	13,448	14,326	16,520	17,296	19,301	18,524
5183	AP	788	241	776	2537	2834	2809	2793	2677	2720	2643	3094	2871	2407	3600	3475	3244	3775
5183	AP	788	207	776	5946	9720	9683	9662	7791	7790	7795	8398	7982	7234	9324	9281	9319	9792
5183	AP	788	190	776	8969	18,348	18,329	18,319	13,513	13,401	13,614	14,032	13,503	12,739	15,230	15,403	16,056	16,106
5183	OA	760	259	975	15,515	8032	8088	8122	7388	7242	7511	13,681	14,974	18,228	7751	8184	9185	8187
5183	OA	774	224	911	21,173	11,450	11,481	11,499	9492	9366	9598	16,940	17,454	18,968	10,388	10,714	11,539	11,405
5183	OA	788	224	847	10,317	5221	5188	5168	4546	4582	4518	8812	8386	7628	5770	5655	5473	6067
5183	PA	760	310	886	1849	1545	1545	1545	1771	1773	1768	2060	2155	2262	2170	2199	2239	1729
5183	PA	760	241	886	12,170	14,068	14,201	14,281	12,011	11,688	12,284	11,618	12,665	15,222	11,949	12,795	14,843	13,395
5183	PA	774	276	847	1655	1999	1989	1984	2091	2110	2075	2793	2757	2602	2699	2665	2589	2640
5183	PA	774	241	847	8045	6351	6352	6352	5695	5663	5722	6913	6972	7064	6590	6697	6966	7052
5183	PA	788	241	807	4274	2834	2809	2793	2677	2720	2643	4036	3770	3226	3600	3475	3244	3775
5793	PA	760	293	863	3114	2667	2673	2676	2842	2825	2854	2592	2726	2914	3308	3398	3574	2949
5793	PA	760	276	863	4382	4617	4638	4651	4572	4514	4620	4025	4278	4733	5054	5263	5717	4950
5793	PA	760	224	863	22,216	24,872	25,168	25,348	19,682	19,012	20,255	15,491	17,004	20,902	18,554	20,151	24,177	21,804
5793	PA	774	259	829	4519	3553	3544	3539	3442	3448	3436	3808	3785	3661	4207	4215	4236	4338
5793	PA	774	207	829	11,123	20,879	20,988	21,055	15,976	15,643	16,261	15,615	16,057	17,339	16,520	17,296	19,301	18,524
5793	PA	774	224	829	11,746	11,450	11,481	11,499	9492	9366	9598	9679	9840	10,235	10,388	10,714	11,539	11,405
5793	PA	788	259	796	2845	1550	1532	1521	1587	1625	1556	2278	2099	1717	2259	2148	1935	2337
5793	PA	788	207	796	10,277	9720	9683	9662	7791	7790	7795	9791	9341	8568	9324	9281	9319	9792
Unknown	PA	760	221	893	34,955	27,912	28,258	28,469	21,750	20,979	22,412	20,816	23,013	28,949	20,282	22,092	26,686	24,039
Unknown	PA	774	221	857	18,583	12,899	12,940	12,965	10,524	10,369	10,656	13,013	13,323	14,184	11,389	11,782	12,779	12,557
Unknown	PA	777	193	850	24,994	30,499	30,675	30,782	21,942	21,427	22,385	25,303	25,984	28,402	22,219	23,336	26,306	24,288
Unknown	PA	788	193	822	15,975	16,137	16,112	16,098	12,090	12,009	12,163	17,522	16,951	16,272	13,793	13,904	14,384	14,544
Unknown	PA	788	179	822	24,951	27,089	27,108	27,121	18,942	18,690	19,164	26,190	25,582	25,340	20,577	21,013	22,414	22,110
Unknown	PA	802	179	786	12,187	12,400	12,298	12,239	9116	9185	9066	16,570	15,044	12,708	11,459	11,129	10,687	11,758
Unknown	PA	802	165	786	18,363	21,500	21,374	21,301	14,689	14,694	14,696	25,338	23,241	20,295	17,527	17,259	17,120	18,490
Unknown	PA	816	165	751	10,122	9811	9669	9585	7061	7210	6945	16,035	13,692	10,228	9733	9122	8157	9935

Case 1: Data Set = All Data, Average TS values, $Q_{C}^{*}$ Determination Method = Arrhenius Plot, $Q_{C}^{*}$ = 284, *u* = 0.0975, *k*_1_ = 12.9.

Case 2: Data Set = All Data, Average TS values, $Q_{C}^{*}$ Determination Method = Correlation Optimization, $Q_{C}^{*}$ = 289, *u* = 0.0971, *k*_1_ = 13.5.

Case 3: Data Set = All Data, Average TS values, $Q_{C}^{*}$ Determination Method = Self-diffusion Activation, $Q_{C}^{*}$ = 292, *u* = 0.0969, *k*_1_ = 13.8.

Case 4: Data Set = All Data, Heat- and processing-specific TS values, $Q_{C}^{*}$ Determination Method = Arrhenius Plot, $Q_{C}^{*}$ = 283, *u* = 0.114, *k*_1_ = 18.4.

Case 5: Data Set = All Data, Heat- and processing-specific TS values, $Q_{C}^{*}$ Determination Method = Correlation Optimization, $Q_{C}^{*}$ = 272, *u* = 0.115, *k*_1_ = 16.3.

Case 6: Data Set = All Data, Heat- and processing-specific TS values, $Q_{C}^{*}$ Determination Method = Self-diffusion Activation, $Q_{C}^{*}$ = 292, *u* = 0.113, *k*_1_ = 20.2.

Case 7: Data Set = Data with *t_r_* < 10,000 h, Average TS values, $Q_{C}^{*}$ Determination Method = Arrhenius Plot, $Q_{C}^{*}$ = 150, *u* = 0.132, *k*_1_ = 3.91.

Case 8: Data Set = Data with *t_r_* < 10,000 h, Average TS values, $Q_{C}^{*}$ Determination Method = Correlation Optimization, $Q_{C}^{*}$ = 196, *u* = 0.126, *k*_1_ = 7.20.

Case 9: Data Set = Data with *t_r_* < 10,000 h, Average TS values, $Q_{C}^{*}$ Determination Method = Self-diffusion Activation, $Q_{C}^{*}$ = 292, *u* = 0.111, *k*_1_ = 19.9.

Case 10: Data Set = Data with *t_r_* < 10,000 h, Heat- and processing-specific TS values, $Q_{C}^{*}$ Determination Method = Arrhenius Plot, $Q_{C}^{*}$ = 207, *u* = 0.126, *k*_1_ = 8.32.

Case 11: Data Set = Data with *t_r_* < 10,000 h, Heat- and processing-specific TS values, $Q_{C}^{*}$ Determination Method = Correlation Optimization, $Q_{C}^{*}$ = 235, *u* = 0.122, *k*_1_ = 11.6.

Case 12: Data Set = Data with *t_r_* < 10,000 h, Heat- and processing-specific TS values, $Q_{C}^{*}$ Determination Method = Self-diffusion Activation, $Q_{C}^{*}$ = 292, *u* = 0.114, *k*_1_ = 20.9.
